# The GRONORUN study: is a graded training program for novice runners effective in preventing running related injuries? Design of a Randomized Controlled Trial

**DOI:** 10.1186/1471-2474-8-24

**Published:** 2007-03-02

**Authors:** Ida Buist, Steef W Bredeweg, Koen APM Lemmink, Gert-Jan Pepping, Johannes Zwerver, Willem van Mechelen, Ron L Diercks

**Affiliations:** 1 University Center for Sport, Exercise and Health, University of Groningen, University Medical Center Groningen, Hanzeplein 1, 9700 RB Groningen, The Netherlands; 2 Center for Sports Medicine, University Medical Center Groningen, Hanzeplein 1, 9700 RB Groningen, The Netherlands; 3 Center for Human Movement Sciences, University of Groningen, University Medical Center Groningen, P.O. Box 196, 9700 AD Groningen, The Netherlands; 4 Department of Public and Occupational Health/EMGO Institute, VU University Medical Center, Van der Boechorststraat 7, 1081 BT Amsterdam, The Netherlands

## Abstract

**Background:**

Running is a popular form of recreational exercise. Beside the positive effects of running on health and fitness, the risk of a running related injury has to be considered. The incidence of injuries in runners is high and varies from 30–79%. However, few intervention studies on prevention of running related injuries have been performed and none of these studies involved novice runners.

**Methods:**

GRONORUN (Groningen Novice Running) is a two armed randomized controlled trial, comparing the effects of two different training programs for novice runners on the incidence of running related injuries. Participants are novice runners, who want to train for a four mile running event. The control group will train according a standard 8 week training program. The intervention group will use a more gradual, 13 week training program which is based on "the ten percent training rule". During the thirteen week follow up participants register information on running and RRI's in an internet based running log. The primary outcome measure is RRI. An injury is defined as a musculoskeletal ailment of the lower extremity or back, causing a restriction of running for at least one week.

**Discussion:**

The GRONORUN trial is the first randomized controlled trial to study a preventive intervention in novice runners. Many different training programs for novice runners are offered, but none are evidence based.

## Background

Worldwide, running is a sport practiced by many individuals to improve cardio-respiratory function, health and well-being [1]. The Royal Dutch Athletics Federation (KNAU) has estimated that around 12.5% of all Dutch people are running on a regular basis, and that the popularity of running events is still growing. The popularity of running positively contributes to increasing levels of physical activity in the population. This is important, because physical inactivity is associated with the development of several chronic diseases, decreased longevity, loss of physical function and weight control [2]. Running is a feasible way for people to become more active. To start with running, just a pair of shoes is needed.

Although running positively contributes to health, there is also the possibility of a running related injury (RRI). The incidence of RRI's injuries in runners at recreational and competitive level is high and varies from 30% to 79% [3-9]. The wide range in incidence is caused by 1) differences in injury definition, 2) time of follow up, 3) differences in population at risk, and 4) differences in methods used to assess RRI, as well as exposure to running. Taking into account the exposure to running, an appropriate way to describe the incidence of RRI's is to calculate the number of RRI's per 1000 hours of running in the population at risk. Injury incidence per exposure varies from 7 to 59 per 1000 hours of running [3-5,10].

Most injuries in runners are overuse injuries of the lower extremity, caused by training errors, that is, running too much, too soon [11]. The exact cause and risk factors of RRI's are still unclear. However, it can be stated that the aetiology of these injuries is multifactorial and diverse. A review by Van Mechelen [12] proposed four factors that have been significantly related to running injuries: a) lack of running experience, b) previous injury, c) running to compete, and d) excessive weekly running distance.

Randomized controlled trials on the effect of interventions for preventing RRI in recreational runners are hard to find. A large amount of the information about the prevention of RRI's is derived from military recruits during basic training [13-20]. A Cochrane review on prevention of injuries in runners showed three categories of preventive strategies: 1) warming-up, cool down and stretching exercises, 2) use of external devices such as shock absorbing insoles, and 3) modification of training schedule [21]. Unfortunately, none of the interventions showed a significant effect in the prevention of RRI's.

Training is required to develop the ability to run. If the stress stimulus of running is optimal, a positive adaptation of structures will take place. An optimal stimulus along with an adequate recovery time will lead to an increase in strength [22]. With the increasing ability to run, the structures ability to handle applied stress also increases.

To minimize the risk of a RRI, an increase of training duration or intensity by no more than 10% is recommended, i.e. the 10% rule [23]. However, so far no studies have examined the effect of such an increase of training load on injury risk in runners.

The GRONORUN trial is designed to examine the effect of a graded training program for novice runners on the incidence of running related injuries. In the current study, we hypothesize that when the human body gets more time for adaptation to running, the incidence of running related injuries will decrease. The objective of the GRONORUN trial is to evaluate the effectiveness of a 13-week graded training program, using the 10% rule, on the incidence of RRI's in a group of novice runners preparing for a four mile run compared to a commonly used 8-week training program. In this article we describe the design of the GRONORUN trial.

## Methods/design

The GROningen NOvice RUNing (GRONORUN) study is a randomized controlled trial (RCT) with a thirteen week follow-up. Participants were randomized into an intervention group (13 weeks training program) or an active control group (8 week training program). Recruitment of participants for the GRONORUN trial took place in May and June 2005 and data collection started in July 2005. The intervention training program started in the second week of July, 13 weeks before the four mile running event, which took place in October 2005.

The study design, procedures and informed consent procedure were approved by the Medical Ethics Committee (Number 2004/285) of the University Medical Center Groningen (UMCG), The Netherlands. All participants provided written informed consent. Guidelines were followed according to the Consort Statement [24].

### Study population

Recruitment was assisted by advertisements in local media to recruit participants who wanted to start a "beginners program" for the Groningen four mile recreational running event. It was not necessary to ultimately participate in the four mile running event itself. Potential participants were sent written information about the study along with a baseline questionnaire and an invitation for an initial interview at the Center for Sports Medicine of the UMCG.

### Inclusion & exclusion criteria

Healthy participants between 18 and 65 years of age, who had no injury of the lower extremity in the three months prior to inclusion and who had not been running on a regular basis in the previous twelve months were eligible for inclusion in the study. Participants were excluded if there were absolute contraindications for vigorous physical activities according to the American College of Sports Medicine [25], or in case of unwillingness to keep a running log.

### Sample size

A power calculation was carried out for the main outcome variable RRI, using a logistic rank survival power analyses. In other studies on novice runners incidence of RRI varies from 29.5 to 58% in a periods of respectively 13 to 28 weeks [3,8]. For the GRONORUN trial we expected an injury incidence of 30%. With a hypothesized 25% reduction of RRI's in the intervention group compared to the control group, a total of 436 runners (2 × 218) were needed for a power of 80% and an alpha of 0.05. The hypothesized reduction was based on clinical relevance, because no other studies on the prevention of RRI in novice runners were found. Assuming an attrition of 15% in the intervention period, a total of 512 (2 × 256) novice runners were needed to detect an effect of the intervention.

### Baseline measurements

#### Questionnaire

The baseline questionnaire consisted of five parts and was sent back by mail in a pre-paid envelope before the initial appointment at the hospital.

Part one covered demographic variables such as name, address, age, gender, and e-mail address.

Information about medical history was collected by the second part of the questionnaire. Conditions related to risk factors for cardiovascular diseases were assessed using a series of questions according to the American College of Sports Medicine [25]. Questions about previous musculoskeletal complaints of the lower extremity and back were assessed per anatomical site. Open-ended questions were used to obtain information about body height (in cm) and body weight (in kg). These self-reported body height and weight data were used to calculate BMI (weight (kg)/height^2^(m)).

Sports participation was assessed in part three by using questions concerning type of sport and mean hours of sports participation. Furthermore, a question on running experience in the past ("Have you ever participated in running on a regular basis?") was used to assess the novelty to running.

Part four consisted of the Jenkins Activity Survey (JAS). The JAS is a tool to indicate type A behaviour [26]. Individuals with a pronounced type A behaviour, also referred to as coronary prone behaviour, are possibly more prone to injury [27]. Type A behaviour is characterized by above average achievement drive, aggressiveness, hostility, impatience, time urgency, and competitiveness [26].

Part five assessed the motivation for running, using a Dutch translated version of the Motivation Of Marathoners Scale (MOMS). The MOMS is an instrument that measures the motives of runners, by means of 56 items distributed across nine scales. Content areas covered include health orientation, weight concern, self-esteem, life meaning, psychological coping, affiliation, recognition, competition, and personal goal achievement. The MOMS was validated by Masters [28], and demonstrated an adequate internal consistency (Cronbach's alpha range .80 to .93), retest reliability (intraclass correlations range .71 to .90), and factorial validity of the scales.

#### Initial interview

During the initial interview at the UMCG all participants were seen by a sports medicine physician. The purpose of the initial interview was to screen for cardiovascular diseases and abnormalities of lower limb and, to ensure that the participants were adequately informed about the study before signing informed consent.

#### Orthopeadic examination

An universal goniometer with arm length 30 cm from axis to tip was used to measure all range of motions with recordings in increments of 1.0°. The internal and external range of motion of the hip was assessed with the participant supine and the tested hip and knee flexed to 90°. Knee flexion and extension ranges of motion were assessed with the participant in supine position. The goniometer was placed on the lateral aspect of the knee, with the axis of the goniometer in line with the greater trochanter and the lateral malleolus. Ankle plantarflexion and dorsiflexion were measured both with the knee fully extended and flexed to 90°. One arm of the goniometer was aligned with the fibular bone and the other with the plantar surface of the foot. Furthermore, the navicular drop was assessed by measuring the change in the height of the navicular tuberosity between a participant sitting with the subtalar joint in neutral position and participant standing, weight bearing with the subtalar joint in relaxed stance, as described by Brody [29]. The navicular drop is a valid method to indicate the amount of foot pronation [30]. Intratester and intertester reliability of this technique is ranging from .73 to .96 [31]. Measurements were made twice for each foot, with results being averaged.

### Randomization

After baseline measurements and informed consent, participants were assigned to the intervention training program or the control training program. To ensure that both training groups were equal in terms of injury risk, a stratified randomization was performed. Participants were stratified for current sporting activities status, previous injury, and gender. Based on sporting activities, there are three categories of novice runners. The first category consists of novice runners who already are participating in a sport in which axial load (i.e., running, walking or jumping) is integrated. The second category is formed by novice runners who already are participating in sporting activities without axial load, like swimming and cycling. The third category is formed by novice runners who did not participate in any sporting activities at baseline measurements. In a study by Macera [6], a 74% increased risk was found in runners with a positive history of previous injuries. Since it is not clear whether the high rate of re-injury is caused by incomplete healing of a previous injury or a biomechanical problem, a differentiation in time is made. A distinction can be made between no previous injury, sustaining injured in the last 12 months before baseline measurements, and sustaining injured more than 12 months before baseline measurements. Eighteen strata were formed by gender, previous injury (no, 3–12 months and > 12 months) and sporting activities (no, with axial load and without axial load). From each stratum participants were allocated to intervention or control group by drawing a sealed opaque envelope. Each stratum box contained equal numbers of control and intervention envelopes.

### Participant flow

**Figure 1:**
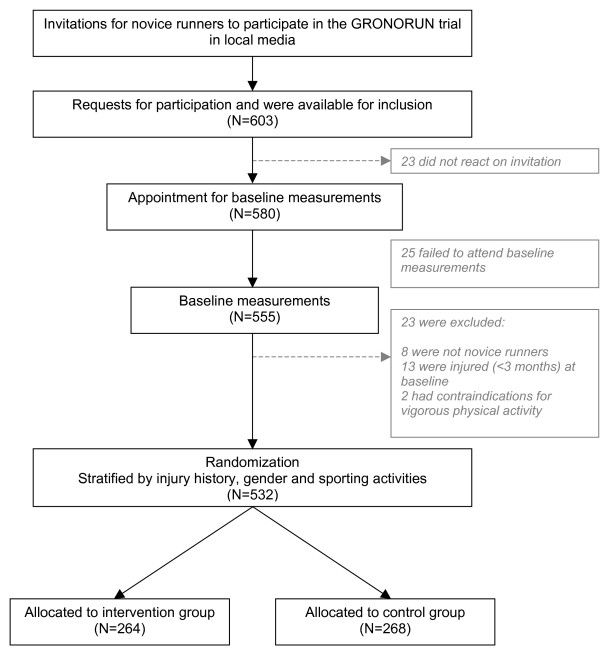
GRONORUN flow chart.

The study design and participants flow are shown in Figure 1. A total of 603 people were interested to participate in the GRONORUN trial and reacted on the call for novice runners. All of those who responded to the advertisements were sent an information package containing: a brochure in which the study protocol was clearly described, a baseline questionnaire, and an appointment at the UMCG. Twenty three did not confirm their appointment for the initial appointment nor sent back the baseline questionnaire. Of those who confirmed the appointment for the initial appointment and sent back the questionnaire (n = 580), twenty five failed to attend the initial appointment. Of 555 persons who visited the UMCG for an initial appointment, 23 were excluded. Reasons for exclusion were: already participating in running (8), musculoskeletal injury of lower extremity or back at baseline (13) and contraindications for vigorous physical activity (2). After baseline measurements and stratification, 532 persons were randomly assigned to the intervention group (n = 264) and to the control group (n = 268).

### Training program

All participants received the same general written and oral information on intensity of running and on warming up and cooling down. Participants were instructed to walk brisk for 5 minutes as a warm up, and 5 minutes as cool down. Given that the best available evidence indicates that stretching before or after exercise does not prevent muscle soreness or injury [32], participants were instructed not to perform stretching exercises before, during or after the training sessions.

The frequency of running was equal in both groups. Each training week, except the last week, that is, the week of the four mile run, consisted of three training sessions represented by a combination of running and walking. Participants were encouraged to run on Monday, Wednesday, and Saturday, and were advised to run at a comfortable pace at which they could converse without breathlessness. Both groups trained individually, without a trainer, on a self-chosen course.

#### Control group: 8 week training program

The novice runners in the control group received a frequently used beginners training program to prepare for a four mile run. The program started 8 weeks before the start of the Groningen four mile run at the level of a run-walk session with a total of 10 minutes of running and 10 minutes of walking (see Table 1).

#### Intervention group: 13 week training program

The intervention group started the 10% rule training program 13 weeks before the start of the Groningen four mile running event (see Table 2). Gradual increase of training load, that is, time of running was 10% per week and the ratio between running and walking was also increasing. The starting point of the program was exactly the same as the start of the program of the control group (i.e., ten minutes of running, interchanged with walking).

### Outcome measures

The primary outcome of the GRONORUN trial is the number of RRI's in both groups. Definition of a RRI in this trial is; running related musculoskeletal ailment of the lower extremity or back, causing a restriction of running for at least one week, that is, three consecutive training sessions.

Information on RRI's and exposure data were collected using an internet based running log. Each of the participants received a study number and a password to enter a personal environment of the internet based training log. After each training week participants had to fill in their running activities, other sport activities and injuries.

Per training session the total minutes of running, total minutes of walking, and injuries were registered. Data on injuries were collected by registering anatomical site of the body and severity of pain. Severity of pain was subdivided in pain without limitation (no RRI), pain that caused a restriction of running (RRI), and running impossible through RRI (RRI). In case of skipping a training session, the reason (RRI, other injury, motivation, illness, or remaining reason) for it was asked. When a "running related injury" was the reason for not running, information on anatomical site and severity was asked. A picture of the lower body was used to assess the anatomical site of the RRI. By clicking on the anatomical site, the area of the RRI was red appointed. When participants did not enter their digital training log after one week, a reminder was sent by email automatically.

### Statistical analyses

To evaluate the success of the randomization, baseline values will be analyzed for differences between intervention group and control group, using a chi-square for categorical data and a student's t-test for numerical data. To analyze the primary outcome (i.e., RRI), the Kaplan-Meier method will be used. Once a participant has a RRI his or her survival time will be terminated. To evaluate the effect of the intervention, a log rank test will be used to compare the Kaplan-Meier curves of the intervention group to the control group. Analyses will be performed following the "intention to treat" principle. Differences will be considered statistically significant at p < 0.05. All analyses will be done using SPSS version 12 (SPSS, Chicago, Illinois).

## Discussion

The GRONORUN trial is the first randomized controlled trial to study the effect of a modification of a training program on RRI's in novice runners. There is a need for well controlled trials about preventive interventions in running populations because of the popularity of running and the high rates of RRI's.

Novice runners are often physically inactive before they start to run. The health benefits in this previously physically inactive group can be high. On the other hand, lack of running experience is one of the risk factors for a RRI [6]. The major reason for discontinuation (drop out) of a running program is injury [33]. Negative experiences, caused by an injury that occurs while training for a running event, have the potential to significantly affect the future physical activity of each individual [34]. It is also known that (fear of) sustaining an injury is associated with failure to start and maintain a physically active lifestyle [34]. So, prevention of injuries in novice runners is important.

On the internet and in running shops different training programs for novice runners are available. Most of the programs are based on expert opinion. There are numerous "experts" and they all have their own opinion of "the best running program", however none of them are based on scientific evidence.

As a result of the GRONORUN trial, valuable information will be gained on training programs for novice runners. With this new information on training programs, it might be possible to reduce the incidence of RRI's in future.

## Competing interests

The author(s) declare that they have no competing interests.

## Authors' contributions

SW conceived of the idea, obtained funding for the study, and developed the intervention. SW and IB developed the design of this trial, and recruited participants. WM provided advice on the study design and contributed to the content of the article. IB is the study investigator, was responsible for data acquisition, and wrote the article. KL and RD are co applicants of the grant. JZ and GJP contributed to the content of the article. All authors read and approved the final manuscript.

## Pre-publication history

The pre-publication history for this paper can be accessed here:



## Figures and Tables

**Table 1:** 8-week-training program for the control group.

	**training 1**			**training 2**			**training 3**			**total**	
	**run**	*walk*	(rep.)	**run**	*walk*	(rep.)	**run**	*walk*	(rep.)	**run**	*walk*

week 1	**1**	*1*	(10)	**1**	*1*	(10)	**1**	*1*	(10)	**30**	*30*
week 2	**4**	*2*	(4)	**4**	*2*	(4)	**4**	*2*	(3)	**46**	*22*
week 3	**6**	*2*	(3)	**12**	*3*	(2)	**6**	*2*	(3)	**60**	*18*
week 4	**6**	*2*	(3)	**7**	*2*	(2)	**6**	*2*	(3)	**50**	*16*
week 5	**10**	*2*	(2)	**17**	*4*	(2)	**10**	*2*	(2)	**74**	*16*
week 6	**15**	*5*	(2)	**20**	*5*	(2)	**20**	*0*	(1)	**90**	*21*
week 7	**40**	*0*	(1)	**30**	*5*	(1)	**25**	*0*	(1)	**95**	*5*
week 8	**30**	*0*	(1)								

The content of each training session is expressed in minutes of running (run), minutes of walking between the running sessions (walk) and number of repetitions (rep.). The right column contains total minutes of running and walking of each week.

**Table 2:** 13-week-training program for the intervention group.

	**training 1**			**training 2**			**training 3**			**total**	
	**run**	*walk*	(rep.)	**run**	*walk*	(rep.)	**run**	*walk*	(rep.)	**run**	*walk*

week 1	**1**	*1*	(10)	**1**	*1*	(10)	**1**	*1*	(10)	**30**	*30*
week 2	**2**	*1,5*	(5)	**2**	*1,5*	(7)	**2**	*1,5*	(7)	**34**	*25,5*
week 3	**3**	*2*	(4)	**3**	*2*	(4)	**3**	*2*	(4)	**36**	*24*
week 4	**4**	*2*	(3)	**4**	*2*	(4)	**4**	*2*	(3)	**40**	*20*
week 5	**4**	*2*	(4)	**4**	*2*	(4)	**4**	*2*	(3)	**44**	*22*
week 6	**6**	*2*	(3)	**6**	*2*	(3)	**6**	*2*	(2)	**48**	*16*
week 7	**6**	*2*	(3)	**9**	*3*	(2)	**6**	*2*	(3)	**54**	*18*
week 8	**6**	*2*	(3)	**6**	*2*	(3)	**10**	*3*	(2)	**56**	*18*
week 9	**8**	*2*	(3)	**12**	*0*	(1)	**14**	*4*	(2)	**64**	*14*
week 10	**10**	*2*	(2)	**16**	*5*	(2)	**10**	*2*	(2)	**72**	*18*
week 11	**15**	*5*	(2)	**30**	*0*	(1)	**20**	*5*	(2)	**80**	*15*
week 12	**30**	*0*	(1)	**20**	*0*	(1)	**40**	*0*	(1)	**90**	*0*
week 13	**30**	*0*	(1)								

The content of each training session is expressed in minutes of running (run), minutes of walking between the running sessions (walk) and number of repetitions (rep.). The right column contains total minutes of running and walking of each week.
